# Case Report: Laparoscopic repair of indirect inguinal hernia combined with ipsilateral giant subcutaneous inguinoperineal lipoma

**DOI:** 10.3389/fmed.2026.1867064

**Published:** 2026-07-23

**Authors:** Yuxuan Mo, Qi Zheng, Binggen Li, Lingjia Tang

**Affiliations:** 1 Department of Gastrointestinal Surgery, Ningbo No. 2 Hospital, Ningbo, China; 2 Department of General Surgery, Nanfang Hospital, Southern Medical University, Guangzhou, Guangdong, China; 3 Department of Geriatrics, Ningbo No. 2 Hospital, Ningbo, China

**Keywords:** giant lipoma, inguinal hernia, laparoscopy, lipoma, subcutaneous lipoma, totally extraperitoneal hernia repair

## Abstract

Lipomas are common benign mesenchymal neoplasms with slow growth, consisting of mature adipocytes, while giant inguinal lipomas are rare because of anatomical limitations. This report describes a 46-year-old woman who presented with a reducible left inguinal mass and an ipsilateral painless giant subcutaneous mass. She was diagnosed with a left indirect inguinal hernia and a separate inguino-labial lipoma superficial to the external oblique aponeurosis, which was not adherent to the inguinal canal, round ligament or canal of Nuck. The coexistence of the two lesions was incidental. The patient underwent combined laparoscopic totally extraperitoneal (TEP) hernia repair and TEP-guided transfascial lipoma excision. Histopathology verified a mature lipoma without atypia or features of liposarcoma. We summarize the clinical manifestations, diagnostic points, anatomy, surgical techniques, pathological results and clinical outcomes of this rare condition, and confirm that single-stage TEP-guided transfascial lipoma excision is a safe and feasible procedure.

## 1 Introduction

Lipoma is a common benign soft tissue tumor composed of mature adipocytes (1). It most frequently occurs in individuals aged 40–60 years and is characterized by slow growth (2). Lipomas are usually round, oval or multilobulated, mobile, and develop into well-encapsulated, circumscribed lesions over time (3). While lipomas may arise in any region containing adipose tissue, giant lipomas in the inguinal region are rare, and the coexistence of an inguinal hernia and a subcutaneous inguino-labial lipoma is extremely uncommon, with limited evidence supporting single-stage minimally invasive treatment. Standard laparoscopic totally extraperitoneal (TEP) inguinal hernia repair is a well-established procedure; however, its extended application for TEP-guided transfascial excision of concurrent subcutaneous lipoma has rarely been reported. In the present case, the two lesions occurred incidentally with no proven causal relationship. We herein report a middle-aged woman with a left inguinal hernia accompanied by a giant subcutaneous lipoma in the ipsilateral perineum, who received successful single-stage combined surgery and achieved favorable clinical outcomes. Meanwhile, we elaborate on the relevant anatomical features, surgical techniques and pathological findings in detail.

## 2 Case presentation

### 2.1 Clinical manifestations, physical examination and preoperative imaging assessment

A 46-year-old woman presented to the hernia clinic of our institution with a reducible mass in the left inguinal region associated with distension and discomfort for 18 months. The mass gradually increased in size over time and caused persistent discomfort that affected her daily activities and quality of life. Focused physical examination revealed a soft, reducible mass approximately 2 cm in diameter at the level of the left inguinal internal ring. The deep ring occlusion test was positive, consistent with indirect inguinal hernia. In addition, a firm, homogeneous, non-tender subcutaneous mass approximately the size of a fist with well-defined margins and poor mobility was palpated lateral to the left labium majus. It was entirely subcutaneous and superficial to the external oblique aponeurosis, did not extend into the inguinal canal, and had no connection to the round ligament or canal of Nuck. No inguinal lymphadenopathy was detected bilaterally.

**Figure 1:**
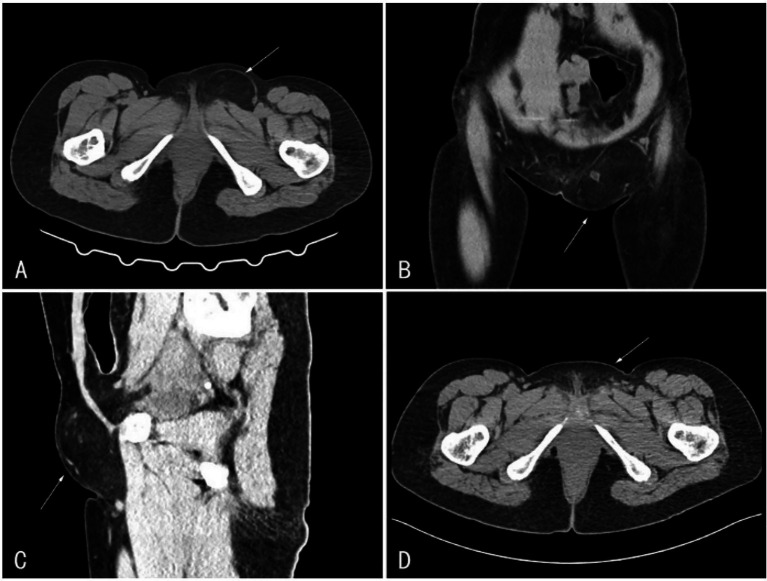
Perioperative CT imaging of the patient. **(A)** Preoperative coronal CT image of the inguinal region demonstrating a subcutaneous lipoma, indicated by the arrow. **(B)** Preoperative three-dimensional reconstructed CT image of the inguinal region showing the subcutaneous lipoma (arrow). **(C)** Preoperative lateral view of the three-dimensional reconstructed CT image of the inguinal region, with the subcutaneous lipoma indicated by the arrow. **(D)** Coronal CT image obtained 3 months postoperatively demonstrating complete resolution of the subcutaneous lipoma, with no evidence of seroma, hematoma, or lipoma recurrence.

Ultrasonography was performed because clinical examination suggested two distinct lesions: a typical reducible inguinal hernia and a separate non-reducible subcutaneous mass, raising suspicion of dual pathology. Ultrasonography demonstrated a hypoechoic lesion measuring 63 × 40 × 20 mm within subcutaneous fat superficial to the external oblique aponeurosis of the left inguinal region, with regular shape, well-defined margins, intact capsule, and internal linear echogenic strands. Color Doppler flow imaging revealed no significant intralesional blood flow, consistent with a diagnosis of lipoma. A three-dimensional spiral computed tomography scan showed a well-circumscribed, extremely low-density mass in the left inguinal region measuring approximately 80 × 60 × 40 mm, located in the subcutaneous plane and separate from the inguinal canal and round ligament structures, with a CT attenuation value of approximately −105 HU, findings suggestive of a lipoma (Figures 1A–C). The patient reported accelerated growth of the mass over the preceding year and the development of new symptoms attributable to the inguinal hernia, prompting a request for surgical management. Given her scar-prone constitution, she strongly expressed a preference for a minimally invasive surgical approach.

### 2.2 Surgical steps

**Figure 2:**
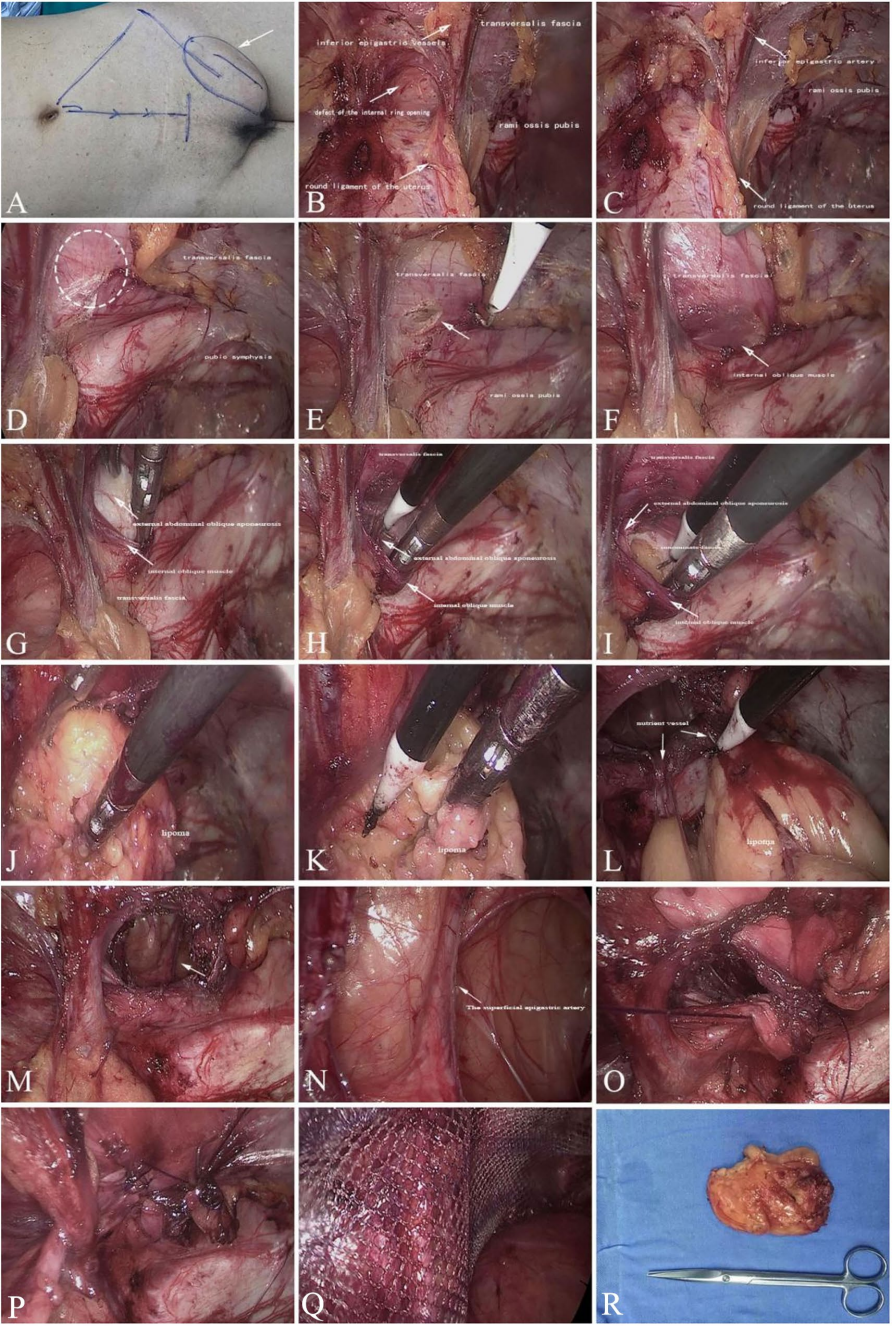
Intraoperative steps of the laparoscopic procedure. **(A)** Surface marking and surgical identification, with the arrow indicating the location of the lipoma. **(B)** Visualization of the internal ring defect following complete dissection of the totally extraperitoneal (TEP) hernia sac. **(C)** Preperitoneal space after completion of pubic muscle space dissection. **(D)** The dashed circle indicates the projected location of the lipoma based on preoperative surface marking prior to incision of the transversalis fascia. **(E)** Sharp incision of the transversalis fascia using an electrocautery hook. **(F)** Exposure of the underlying internal oblique muscle following division of the transversalis fascia (arrow). **(G)** Blunt separation of the internal oblique muscle, revealing the underlying white external oblique aponeurosis. **(H)** Sharp incision of the external oblique aponeurosis with an electrocautery hook. **(I)** Exposure and sharp incision of the unnamed fascia beneath the external oblique aponeurosis, revealing subcutaneous fat. **(J)** Blunt dissection of the lipoma from surrounding loose adhesions. **(K)** Sharp dissection of dense adhesions surrounding the lipoma. **(L)** Identification and division of the nourishing blood vessels supplying the lipoma using an electrocautery hook. **(M)** Large residual cavity following complete removal of the lipoma from the subcutaneous layer (arrow). **(N)** Laparoscopic inspection of the cavity to exclude active bleeding and residual tumor tissue; the arrow indicates a superficial abdominal wall artery. **(O)** Continuous full-thickness closure of the fascial defect using a 3–0 barbed suture. **(P)** Placement of the prosthetic mesh covering the pubic muscle space. **(Q)** Gross appearance of the excised lipoma specimen. **(R)** Final appearance of the skin closure.

The patient received prophylactic intravenous cefazolin 30 min before incision. The patient underwent laparoscopic totally extraperitoneal (TEP) inguinal hernia repair combined with laparoscopic excision of the lipoma. Preoperative surface marking was performed to locate the lipoma (Figure 2A). Intraoperative exploration identified a defect measuring approximately 2 cm in diameter at the left inguinal internal ring (Figure 2B). The TEP hernia repair was performed in strict accordance with established surgical guidelines (4), including standardized port placement, dissection of the hernia sac, management of the round ligament, dissection of the pubic muscle space, and placement of the prosthetic mesh (Figure 2C). Because these steps followed routine and standardized procedures, they are not described in further detail.

After completion of the pubic muscle space dissection, laparoscopic excision of the lipoma was performed. A transverse incision of approximately 5 cm in length was made in the transversalis fascia using an electrocautery hook. This incision was located lateral to the conjoint tendon and did not include or damage the conjoint tendon. A non-traumatic clamp was used to bluntly separate the underlying abdominal oblique muscle, allowing exposure of the external oblique aponeurosis. Subsequent incision of the unnamed fascia enabled visualization of the lipoma capsule. Combined sharp and blunt dissection was employed to separate the tumor from surrounding tissues. Dense adhesions were carefully divided, and nourishing vessels were precisely identified and divided. Under laparoscopic magnification, the ilioinguinal nerve was clearly identified in the aponeurotic and subcutaneous layers. All dissection was kept away from the nerve course to avoid traction or thermal injury. After removal, the laparoscope was introduced into the subcutaneous cavity to confirm no residual tumor or active bleeding. The transversalis fascia and adjacent aponeurotic layers were closed using continuous full-thickness 3–0 barbed suture. A polypropylene mesh measuring 10 × 15 cm was trimmed and placed to fully cover the myopectineal orifice with overlap ≥4 cm beyond the transversalis fascia incision. The excised lipoma was removed through the umbilical port. No surgical drain was inserted because meticulous hemostasis was achieved, dead space was completely closed, and minimal postoperative fluid accumulation was expected. Total operation duration was 85 min, and estimated blood loss was <10 mL (Figures 2D–R).

### 2.3 Specimen and pathology

**Figure 3:**
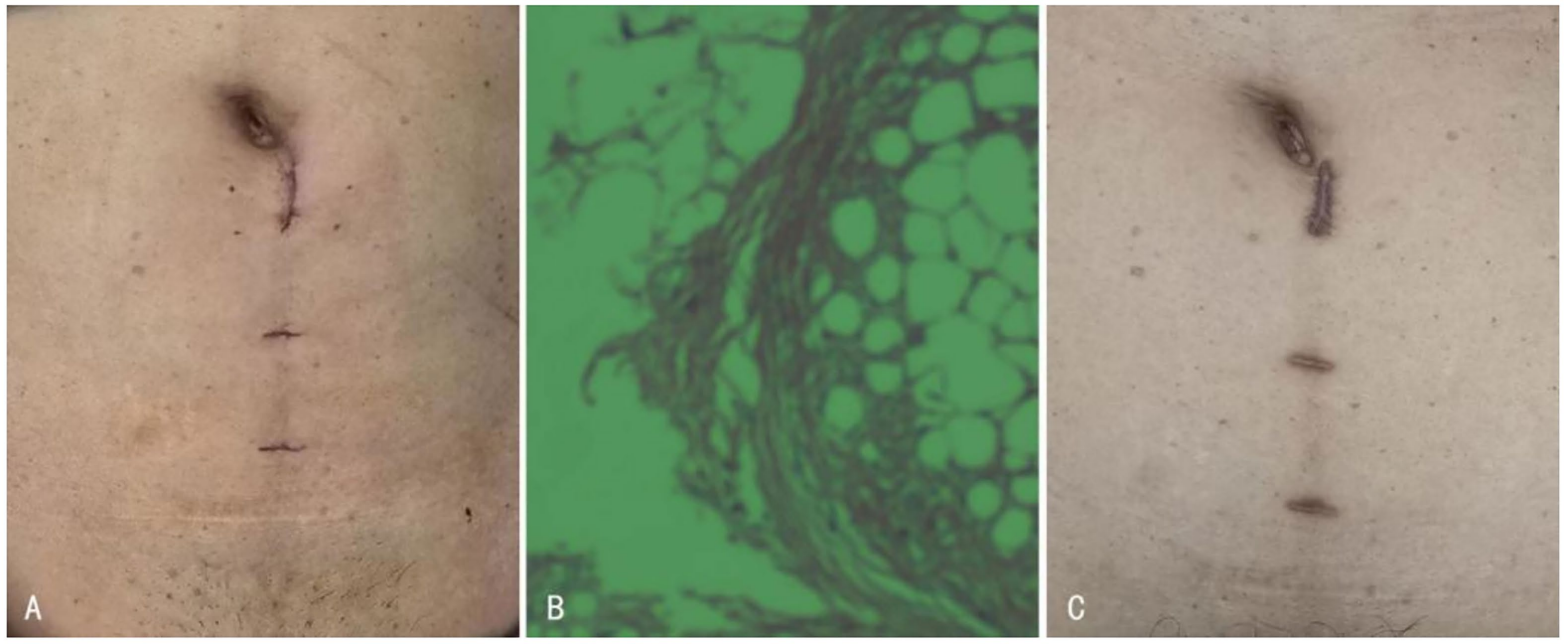
Perioperative wound appearance and pathological findings. **(A)** Wound appearance on postoperative day 1. **(B)** Histopathological examination of the excised specimen confirming the diagnosis of lipoma. **(C)** Development of significant scar hyperplasia at the laparoscopic port site 6 months after surgery.

Gross dimensions: 82 × 62 × 42 mm; weight: 54 g. Histopathological diagnosis: mature lipoma. No cellular atypia, no lipoblasts, no necrosis, and no increased mitotic activity. Thin and sparse fibrous septa were noted. There were no morphological features suggestive of atypical lipomatous tumor/well-differentiated liposarcoma. MDM2/CDK4 immunostaining was not indicated (Figure 3B).

### 2.4 Postoperative recovery and follow-up

Postoperatively, the patient reported mild pain, with a resting numerical rating scale (NRS) score of 2, and was able to ambulate and resume a liquid diet 4 h after surgery. On postoperative day 1, the surgical dressing was changed, and the patient was discharged without complications (Figure 3A).

The patient was followed at 7 days, 1 month, 6 months, and 1 year. CT at 3 months showed no subcutaneous effusion or complications (Figure 1D). No chronic pain was reported. At 6 months, mild port-site scar hyperplasia was observed (Figure 3C). At 1-year follow-up, no hernia or lipoma recurrence was detected.

## 3 Discussion

Lipoma is a highly prevalent benign tumor in clinical practice, with an estimated incidence of approximately 2.1/1,000 (5), and its etiology remains incompletely understood. Lipomas may arise in any anatomical region containing adipose tissue and exhibit substantial variability in size, although the vast majority are small and weigh only a few grams (6). At present, there is no universally accepted definition of giant lipomas. Hawary et al. defined lipomas with a diameter greater than 5 cm and weight exceeding 500 g as large lipomas (7), whereas Uscilowska et al. proposed that lesions exceeding 10 cm in diameter or 1,000 g in weight should be classified as giant lipomas (8).

Large lipomas may cause not only local contour deformities but also sustained compression of adjacent structures, resulting in corresponding clinical symptoms. Previous studies have suggested that chronic pressure exerted by lipomas on local neural structures may represent a risk factor for the development of inguinal hernias (9, 10). Whether lipomas possess malignant potential or may progress to liposarcoma remains a topic of ongoing debate (11). On the basis of these considerations, surgical intervention is commonly recommended for patients with lipomas associated with compressive symptoms or significant cosmetic concerns. Imaging modalities, including ultrasonography, multislice spiral CT, and magnetic resonance imaging, allow accurate delineation of tumor size, margins, and anatomical relationships with surrounding tissues, thereby providing reliable support for preoperative diagnosis and individualized surgical planning (12).

Surgical excision remains the treatment of choice for lipomas. Conventional open resection enables complete removal under direct visualization and offers the advantages of short operative time and reliable efficacy; however, it is often associated with large incisions, prominent postoperative scarring, and local tissue depression, leading to suboptimal cosmetic outcomes (13). Liposuction has been explored as an alternative technique to minimize incision size and improve cosmetic results, but it is a blind procedure that carries a risk of injury to adjacent blood vessels and nerves (14). In addition, liposuction frequently fails to completely remove the lipoma capsule, resulting in a higher likelihood of residual tumor and postoperative recurrence (15), and may be complicated by skin depression, local numbness, sensory disturbances, and pigmentation changes (16, 17). Moreover, liposuction equipment is largely confined to plastic surgery settings, limiting its broader clinical applicability. To better illustrate the spectrum of surgical strategies across different anatomical locations and their corresponding diagnostic challenges, we summarized the relevant literature in Table 1.

**Table 1:** Summary of relevant literature on lipoma surgical strategies across anatomical locations and associated diagnostic challenges.

No.	Authors and year	Location	Core research content/key findings	Treatment method mentioned	Journal	Reference
1	Lanng C et al., 2004 (1)	Breast	Breast lipoma is easy to misdiagnose; differential diagnosis difficulty with other breast masses	Conventional surgical resection	BREAST	(1)
2	Slavchev SA et al., 2017 (2)	Child forearm	Rare deep giant lipoma in pediatric upper limb; deep location increases surgical exposure difficulty	Open surgical excision	Hand Surgery	(2)
3	Ramírez-Montaño L et al., 2013 (3)	Breast	Clinical features and diagnosis of massive breast lipoma; distinguish from breast fibroadenoma and carcinoma	Open resection	Archives of Plastic Surgery (APS)	(3)
4	Bittner R et al., 2012 (4)	Inguinal region	International consensus guidelines for TAPP/TEP hernia repair; provides reference for groin mass differential diagnosis (lipoma vs. hernia)	Laparoscopic/endoscopic hernia repair	Surgical Endoscopy and Other Interventional Techniques	(4)
5	Silistreli K et al., 2005 (5)	Cutaneous whole body	Literature review + 4 clinical cases; summarize selection criteria of treatment for large superficial lipomas	Open surgery, liposuction-assisted excision	British Journal of Plastic Surgery	(5)
6	Hakim E et al., 1994 (6)	Multiple soft tissue sites	Summarize clinical manifestations, imaging features and surgical risks of ultra-large lipomas	Complete surgical enucleation	Plastic & Reconstructive Surgery	(6)
7	Hawary MB et al., 1999 (7)	Breast	Differential diagnosis of large breast masses; lipoma is a benign giant breast tumor with distinct imaging features	Surgical resection for definite diagnosis and treatment	Annals of Saudi Medicine	(7)
8	Uscilowska E et al., 2019 (8)	Perianal	Rare traumatic para-anal lipoma; case report + literature review on trauma-induced lipoma pathogenesis	Local surgical excision	Acta Chirurgica Belgica	(8)
9	Parikh RN et al., 2020 (9)	Groin	Large intermuscular lipoma mimicking inguinal hernia; key points for preoperative differential diagnosis	Surgical excision to distinguish from hernia	Cureus	(9)
10	Saha M, 2015 (10)	Abdominal wall (Spigelian region)	4-year-old child case; intermuscular lipoma easily confused with abdominal wall hernia in children	Exploratory surgical resection	Journal of Indian Association of Pediatric Surgeons	(10)
11	Prasad S et al., 2012 (11)	Pleura	Rare intrathoracic lipoma; verify safety and efficacy of thoracoscopic minimally invasive surgery	Video-assisted thoracoscopic surgery (VATS)	Journal of Minimal Access Surgery	(11)
12	Li Y et al., 2011 (12)	Breast	Chinese case report; summarize clinical, imaging and pathological features of giant breast lipoma	Complete surgical excision	Clinical Breast Cancer	(12)
13	Mustoe TA, 1999 (13)	General plastic surgery field	General review of emerging minimally invasive techniques in soft tissue tumor surgery	Liposuction, endoscopic minimally invasive surgery	Archives of Surgery (Arch Surg)	(13)
14	Takeuchi M et al., 1997 (14)	Back, shoulder	Early endoscopic minimally invasive technique for deep trunk lipoma; small hidden incision via axillary approach	Endoscopic-assisted transaxillary excision	Annals of Plastic Surgery (Aun Plast Surg)	(14)
15	Sanchez MR et al., 1993 (15)	Cutaneous soft tissue	Early systematic summary of giant lipoma epidemiology, clinical presentation and complications	Open surgical resection	Journal of the American Academy of Dermatology	(15)
16	Karen HC, 1995 (16)	Cervicofacial region	Liposuction auxiliary technique reduces incision scar for head and neck lipomas	Liposuction-assisted excision	Otolaryngology–Head and Neck Surgery	(16)
17	Wilhelmi BJ et al., 1999 (17)	Face	Liposuction achieves cosmetic advantages for small superficial facial lipomas compared with open cut	Liposuction minimal access removal	Plastic & Reconstructive Surgery	(17)
18	Pyon J et al., 2008 (18)	Cheek	Endoscopic minimally invasive surgery avoids obvious facial scar for cheek lipoma	Pure endoscopic excision	Annals of Plastic Surgery	(18)

To date, there have been no reports describing complete laparoscopic excision of lipomas. The principal indications for this approach include patients with high expectations for postoperative cosmetic outcomes and those requiring simultaneous surgical treatment for other conditions in the same anatomical region. In the present case, the patient had a left inguinal hernia concomitant with a giant lipoma in the ipsilateral inguinal region and, because of a scar-prone constitution, strongly preferred a minimally invasive strategy. Accordingly, laparoscopic totally extraperitoneal hernia repair combined with lipoma excision was performed under general anesthesia, enabling simultaneous treatment of both lesions through a single minimally invasive approach while optimizing both therapeutic efficacy and cosmetic outcomes. Laparoscopic surgery provides high-definition magnified visualization, enhanced anatomical recognition, minimal tissue trauma, rapid postoperative recovery, and a low complication rate, and has become the standard approach for hernia repair. Importantly, the application of laparoscopic techniques is not limited to natural body cavities, as meticulous dissection can create effective operative spaces even in anatomically confined regions (18).

Subcutaneous lipomas pose particular challenges for laparoscopic management because they lack a natural operative cavity, are surrounded by critical vascular and neural structures, and are located within a restricted working space. In addition, the longitudinal port distribution required for laparoscopic TEP procedures predisposes surgeons to the “chopstick effect,” necessitating advanced bimanual coordination and potentially prolonging operative time. Nevertheless, the present case demonstrates that laparoscopic TEP hernia repair combined with lipoma excision offers clear clinical advantages, achieving an effective integration of minimally invasive principles, therapeutic efficacy, and practical clinical application. The key advantages of this approach are summarized as follows:

1. Comprehensive minimally invasive approach addressing cosmetic considerations in scar-prone patients: This combined procedure achieves both inguinal hernia repair and lipoma excision through a single laparoscopic approach, requiring only enlargement of the umbilical observation port for specimen retrieval. This strategy avoids multiple incisions and substantially reduces the risk of postoperative scar hyperplasia. Laparoscopic minimally invasive surgery minimizes iatrogenic injury to the abdominal wall musculature and vascular structures, resulting in mild postoperative pain and rapid recovery, thereby fully addressing the patient’s preference for minimally invasive treatment while balancing surgical efficacy and esthetic outcomes.
2. Single-stage management of dual lesions, improving diagnostic and therapeutic efficiency: Simultaneous repair of the inguinal hernia and excision of the giant lipoma in a single operative session obviates the need for repeated anesthesia, multiple hospital admissions, and staged postoperative recoveries, thereby significantly shortening the overall treatment course. This approach allows concurrent relief of hernia-related bulging and lipoma-associated compression symptoms, leading to rapid improvement in quality of life while reducing both medical resource utilization and economic burden.
3. Improved precision and safety through enhanced laparoscopic visualization: High-definition, magnified laparoscopic visualization permits clear identification of the pubic muscle space, lipoma capsule, and adjacent vascular and neural structures. This facilitates accurate mesh coverage of the pubic muscle space, thereby reducing the risk of hernia recurrence, while also enabling complete excision of the lipoma capsule, minimizing residual tumor tissue and fundamentally lowering the risk of lipoma recurrence.
4. Streamlined surgical workflow with reduced secondary tissue injury: The operative sequence of “hernia repair first, followed by lipoma excision,” takes advantage of the already dissected pubic muscle space created during hernia repair to extend the operative field and expose the lipoma capsule without establishing additional surgical planes. This approach limits repeated dissection and unnecessary tissue manipulation, thereby reducing secondary tissue injury and lowering the incidence of postoperative complications.

A limitation of this approach is that it violates both the posterior preperitoneal plane (TEP) and anterior subcutaneous plane. To minimize hernia recurrence, we used complete dissection of the myopectineal orifice, large mesh with ≥4 cm overlap, and secure fascial closure. In case of future hernia recurrence, surgical management should utilize virgin extraperitoneal planes or a contralateral approach; an open anterior approach may be used if needed.

The cosmetic benefit of multiple keyhole scars over a single inguinal incision is patient-specific: this patient had a scar-prone constitution, in whom groin incisions frequently result in severe hypertrophic scarring, while laparoscopic port-site scars are smaller and more concealable.

Meticulous identification and preservation of the ilioinguinal nerve is critical to prevent chronic inguinal pain and ensure reproducibility of this technique.

## 4 Conclusion

Laparoscopic TEP inguinal hernia repair combined with subcutaneous inguinal–perineal giant lipoma excision is a safe, feasible, and effective minimally invasive strategy for patients with concurrent dual lesions. This approach integrates therapeutic efficacy, safety, and cosmetic outcomes, especially for scar-prone patients.

## Data Availability

The original contributions presented in the study are included in the article/Supplementary material, further inquiries can be directed to the corresponding author/s.
